# The Temporal Effects of Acute Exercise on Episodic Memory Function: Systematic Review with Meta-Analysis

**DOI:** 10.3390/brainsci9040087

**Published:** 2019-04-18

**Authors:** Paul D. Loprinzi, Jeremiah Blough, Lindsay Crawford, Seungho Ryu, Liye Zou, Hong Li

**Affiliations:** 1 Exercise & Memory Laboratory, Department of Health, Exercise Science and Recreation Management, The University of Mississippi, University, MS 38677, USA; jblough@go.olemiss.edu (J.B.); lcrawfor@go.olemiss.edu (L.C.); sryu2@go.olemiss.edu (S.R.); 2 Lifestyle (Mind-Body Movement) Research Center, College of Sport Science, Shenzhen University, Shenzhen 518060, China; liyezou123@gmail.com; 3 Shenzhen Key Laboratory of Affective and Social Cognitive Science, College of Psychology and Sociology, Shenzhen University, Shenzhen 518060, China; 4 Shenzhen Institute of Neuroscience, Shenzhen 518057, China

**Keywords:** cognition, memory function, physical activity

## Abstract

**Background:** Accumulating research demonstrates that the timing of exercise plays an important role in influencing episodic memory. However, we have a limited understanding as to the factors that moderate this temporal effect. Thus, the purpose of this systematic review with meta-analysis was to evaluate the effects of study characteristics (e.g., exercise modality, intensity and duration of acute exercise) and participant attributes (e.g., age, sex) across each of the temporal periods of acute exercise on episodic memory (i.e., acute exercise occurring before memory encoding, and during memory encoding, early consolidation, and late consolidation). **Methods:** The following databases were used for our computerized searches: Embase/PubMed, Web of Science, Google Scholar, Sports Discus and PsychInfo. Studies were included if they: (1) Employed an experimental design with a comparison to a control group/visit, (2) included human participants, (3) evaluated exercise as the independent variable, (4) employed an acute bout of exercise (defined as a single bout of exercise), (5) evaluated episodic memory as the outcome variable (defined as the retrospective recall of information either in a spatial or temporal manner), and (6) provided sufficient data (e.g., mean, SD, and sample size) for a pooled effect size estimate. **Results:** In total, 25 articles met our inclusionary criteria and were meta-analyzed. Acute exercise occurring before memory encoding (d = 0.11, 95% CI: −0.01, 0.23, *p* = 0.08), during early memory consolidation (d = 0.47, 95% CI: 0.28, 0.67; *p* < 0.001) and during late memory consolidation (d = 1.05, 95% CI: 0.32, 1.78; *p* = 0.005) enhanced episodic memory function. Conversely, acute exercise occurring during memory encoding had a negative effect on episodic memory (d = −0.12, 95% CI: −0.22, −0.02; *p* = 0.02). Various study designs and participant characteristics moderated the temporal effects of acute exercise on episodic memory function. For example, vigorous-intensity acute exercise, and acute exercise among young adults, had greater effects when the acute bout of exercise occurred before memory encoding or during the early memory consolidation period. **Conclusions:** The timing of acute exercise plays an important role in the exercise-memory interaction. Various exercise- and participant-related characteristics moderate this temporal relationship.

## 1. Introduction

Episodic memory refers to the retrospective recall of information from a spatial-temporal context [1]. That is, retrieving previously encoded information based on its place and location in time. Episodic memory function is critical for communicative behavior [2], is predictive of longevity [3], and the deterioration of episodic memory is associated with several neurocognitive and neuropsychiatric disorders [4]. Thus, identification of behaviors that enhance episodic memory is a worthwhile endeavor.

Accumulating research demonstrates that both acute and chronic exercise behavior can subserve cognitive function, including episodic memory [5,6,7,8,9,10,11,12,13,14,15,16,17,18,19,20,21,22,23,24,25,26,27]. Of interest to this review are the effects of acute exercise (vs. chronic exercise) on episodic memory. This tailored focus on acute exercise is justified, as the mechanisms through which acute and chronic exercise influence episodic memory is distinct (discussed elsewhere [28,29]). Recent excellent reviews (e.g., see Roig et al. [14,15]) have qualitatively and quantitatively summarized the effects of acute exercise on memory function. These reviews, as well as other reviews [16,30], have demonstrated that acute exercise has small-to-moderate effects on enhancing memory, and that these effects may, in part, be moderated by key study characteristics (e.g., timing of exercise and the memory assessment, exercise modality, intensity and duration of exercise) and participant attributes (e.g., age, sex).

For example, the timing of the exercise and memory stimulus plays a critical role in the exercise-memory interaction [14,15]. Various temporal periods have been evaluated in recent work on this topic, including acute exercise occurring before memory encoding, during memory encoding, and during the early and late phases of memory consolidation. Accumulating research suggests that when the acute bout of exercise occurs shortly before the memory task, episodic memory may be enhanced [31,32,33,34]. As we have discussed elsewhere [29], this may be a result of acute exercise-induced alterations in long-term potentiation (LTP), or the functional connectivity of communicating neurons.

Although there is accumulated research demonstrating that the timing of exercise plays an important role in influencing episodic memory, we have a limited understanding as to whether this temporal effect of acute exercise on episodic memory is influenced by study characteristics (e.g., exercise modality, intensity and duration of acute exercise) and participant attributes (e.g., age, sex). Such an effect is plausible for several reasons [35]. These characteristics appear to play a moderating role on their own (while not considering the temporal effects of acute exercise on memory). For example, we recently demonstrated that the vigorous-intensity, acute exercise has greater effects in enhancing episodic memory when compared to the lower-intensity, acute exercise [34]. However, it is plausible that vigorous-intensity, acute exercise would have a differential effect on episodic memory based on the timing of the acute bout of exercise and the memory stimulus. For example, acute, vigorous-intensity exercise may enhance memory when it occurs prior to memory encoding (via LTP) and may also enhance long-term memory if it occurs during the consolidation period (e.g., vigorous-intensity, acute exercise-induced increases in neurotrophins may help stabilize the memory trace) [36]. However, vigorous-intensity, acute exercise may have a negative effect on episodic memory if occurring during memory encoding, as this higher-intensity bout of exercise may induce a transient hypofrontality effect and mental fatigue [37,38], which in turn, may reduce memory encoding.

Regarding participant attributes, other reviews demonstrate that, for example, young adults (vs. older adults) appear to have greater memory-enhancing effects from acute exercise [15]. However, it is uncertain as to whether these (exercise intensity, age) and other characteristics (e.g., sex) consistently influence the acute exercise-memory interaction across all exercise-memory temporal periods. Thus, the purpose of this systematic review with meta-analysis was to evaluate the effects of study characteristics (e.g., exercise modality, intensity and duration of acute exercise) and participant attributes (e.g., age, sex) across each of the temporal periods of acute exercise on episodic memory (i.e., acute exercise occurring before memory encoding, and during memory encoding, early consolidation, and late consolidation). We hypothesize that the acute exercise occurring before memory encoding and during early and late consolidation will enhance episodic memory, whereas acute exercise occurring during memory encoding will impair episodic memory. However, of central focus of this review, we hypothesize that key moderators (e.g., vigorous-intensity exercise, age) will influence this temporal relationship between acute exercise and episodic memory function. The identification of potential moderators of this temporal relationship should aid in the design of future projects on this topic as well as, even within one temporal period (e.g., acute exercise before memory encoding), demonstrate the complexity of the acute exercise-memory interaction.

## 2. Methods

### 2.1. Data Sources and Search Strategy

The following databases were used for our computerized searches: Embase/PubMed, Web of Science, Google Scholar, Sports Discus, and PsychInfo [39]. Articles were retrieved from inception to 14 February 2019. The search terms, including their combinations, were: Physical activity, exercise, physical exercise, cognition, memory, episodic memory, and temporality.

### 2.2. Study Selection

The literature searches were performed independently by two separate authors and comparisons were made to determine the number of eligible studies. Consensus was reached from these two independent reviews. Upon performing the computerized searches, the article titles and abstracts were reviewed to identify potentially relevant articles. Articles appearing to meet the inclusionary criteria were retrieved and reviewed at the full text level.

### 2.3. Inclusionary Criteria

Studies were included if they: (1) Employed an experimental design with a comparison to a control group/visit, (2) included human participants, (3) evaluated exercise as the independent variable, (4) employed an acute bout of exercise (defined as a single bout of exercise), (5) evaluated episodic memory as the outcome variable (defined as the retrospective recall of information either in a spatial or temporal manner), and (6) provided sufficient data (e.g., mean, SD, and sample size) for an effect size estimate.

### 2.4. Methodological Quality of Evaluated Studies

Two authors independently evaluated the methodological quality of the included studies. When disagreements between the two reviewers occurred, they discussed the discrepant results together and reached a consensus. The methodological quality of each included study was evaluated using the Physiotherapy Evidence Database (PEDro) scale. This scale is based on 11 items to assess study rigor, including: Eligibility criteria, random allocation, concealed allocation, baseline comparability, blinded subjects, blinded therapists, blinded assessors, follow-up, intention-to-treat, between group analysis, and outcome point estimates. We adapted the PEDro scale to fit our study topic. Of the 11 items, we removed the eligibility criteria item because this focuses more on external validity as opposed to internal validity. Further, blinded subjects and blinded therapists were removed because it is not feasible to blind the subject/researcher for an exercise protocol. Further, follow-up and intention-to-treat items were removed because of the acute exercise paradigm employed in our evaluated experiments. Thus, we retained and evaluated the following items: Random allocation, concealed allocation, baseline comparability, blinded assessors, between group analysis, and outcome point estimates. In addition to these six items, we added an additional internal validity item, including whether studies reported an objective measure of exercise intensity (e.g., heart rate). In total, seven items were evaluated. Studies that met the evaluated criteria were given a point (seven points maximum), with a greater score indicative of higher methodological quality. Points were only awarded if the criterion was clearly satisfied in the paper. Given that some of the studies employed a within-subject design, these studies were automatically awarded a point for the baseline comparability item.

### 2.5. Data Extraction of Included Studies

Detailed information from each of the included studies was extracted, including the following information: Author, sample characteristics, study design, exercise temporality (i.e., when the exercise took place in reference to the memory assessment), exercise protocol, memory assessment, and results.

### 2.6. Categorization of Temporal Period

We evaluated four temporal periods regarding the timing of exercise and the memory assessment. These included: (1) Exercise before memory encoding vs. control, (2) exercise during memory encoding vs. control, (3) exercise during early consolidation vs. control, and (4) exercise during late consolidation vs. control.

The temporal period of ‘exercise before memory encoding’ was defined as the acute bout of exercise occurring prior to encoding the memory stimuli. We defined ‘exercise during memory encoding’ if the acute bout of exercise occurred while encoding the memory stimuli. Early consolidation was considered within the first four h post memory encoding, whereas late consolidation was defined as four or more h after memory encoding. This specific threshold of four h was utilized as the two studies evaluating the effects of exercise during the late consolidation period employed this time frame [19,40].

### 2.7. Categorization of Moderators

The evaluated moderators included age, sex, race-ethnicity, memory type, exercise intensity, exercise duration, and exercise modality. We were not able to evaluate the moderation effects of cardiorespiratory fitness, as too few studies evaluated this parameter. The evaluated moderators were chosen as they have been shown to influence episodic memory [15,30,41]. Notably, however, not all of these moderators could be evaluated for each of the temporal periods. Moderation analyses were computed when at least two studies provided data for the moderation analysis.

Age was categorized as young-adult (18–24 years), adult (25–44 years), middle-age (45–60 years), and older adults (>60 years) [15]. The sex moderation analyses were evaluated for males, females, mixed samples, and predominately male/female. Predominately male/female was defined as a study including >71% of a particular sex [42]. Race-ethnicity was defined as non-Hispanic white and other. For the episodic memory type, we evaluated both short-term and long-term episodic memory, with the latter defined as a delayed period >2-min [15]. Exercise-intensity was based on thresholds suggested by the American College of Sports Medicine [43]. For example, based on maximum heart rate estimates, light, moderate and vigorous-intensity exercise, respectively, were defined as <64%, 64%–76%, and >76%. Exercise duration was defined as short duration (<20 min), medium (20–40 min), and long duration (>40 min) [15]. Lastly, exercise modality was defined as treadmill-based walking/running or cycling.

### 2.8. Data Synthesis

The Comprehensive Meta-Analysis software (Version 3, Biostat, NJ, USA) was used to calculate effect sizes (Cohen’s d) and 95% CI, employing a random-effects model. The weighted mean effect size (Cohen’s d) and 95% CI were calculated using the inverse variance weighting method. Effect size estimates were evaluated for each of the above-mentioned moderators for each temporal period. The degree of heterogeneity of the effect sizes was evaluated with the Cochran’s *Q*-statistic. Egger’s regression test was used to evaluate the potential publication bias.

## 3. Results

### 3.1. Retrieved Articles

**Figure 1:**
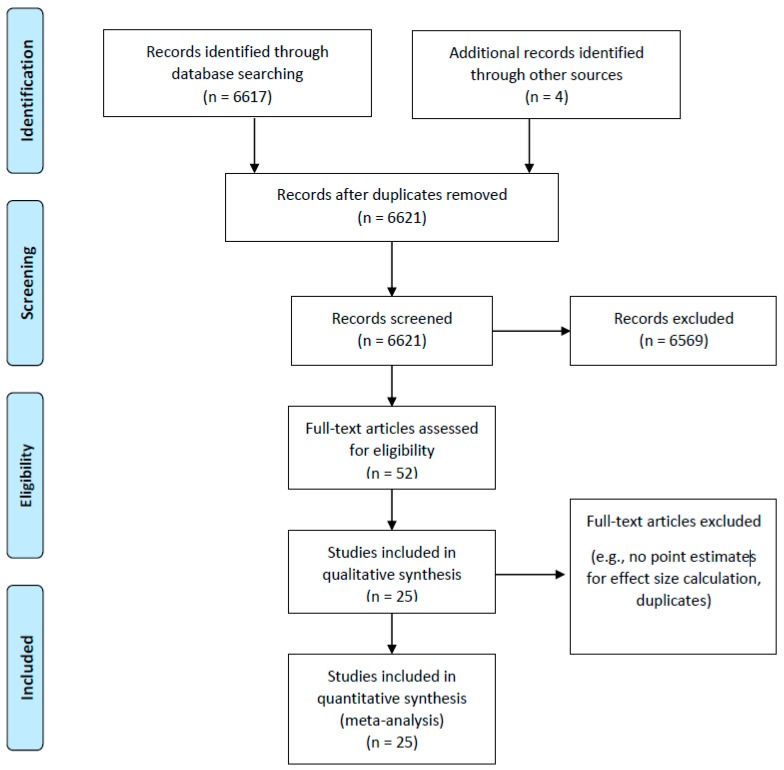
Flow chart of article retrieval.

Figure 1 displays the flow chart of the article retrieval process. The computerized searches identified 6621 articles. Among the 6621 articles, 6569 were excluded and 52 full text articles were reviewed. Among these 52 articles, five were duplicates and 22 were ineligible [5,24,25,26,44,45,46,47,48,49,50,51,52,53,54,55,56,57,58,59,60,61] as they did not provide enough data for an effect size calculation. Thus, in total, 25 articles met our inclusionary criteria and were eligible for the quantitative meta-analysis.

### 3.2. Study Quality

The methodological quality of the studies is shown in Table 1. On a scale from 0–7, the mean score was 4.72 (SD = 0.79). For the random allocation (between-subject design) or counterbalancing (within-subject design) item, 24 of the 25 studies (96.0%) satisfied this criterion. Similarly, the majority of studies (*N* = 21; 84.0%) employed a baseline comparison assessment, objective measure of exercise intensity (*N* = 22; 88.0%), between/within-group analysis (*N* = 25; 100%), and outcome point estimate (*N* = 25; 100%). However, only one study (4.0%) provided sufficient details on whether allocation concealment was employed, and zero studies (0.0%) blinded the assessors to the outcome.

### 3.3. Article Synthesis 

Details on the study characteristics are displayed in Table 2 (extraction table). Studies ranged from young adults (18 years) to older adults (84 years). Sample sizes ranged from 10 to 352 participants. Among the 25 studies, 17 (68%) employed a between-subject design. The acute exercise protocols varied, ranging from an isokinetic resistance exercise, to a 2-min run, to a 60-min brisk walk. Common memory assessments included word list formats (e.g., CVLT, California Verbal Learning Test; RAVLT, Rey Auditory Verbal Learning Test), but other episodic memory assessments included, for example, paragraph recalls, image recognition, and recall of visually observed film stimuli. 

### 3.4. Article Reference

There were too many effect sizes to report in a standard forest plot (as noted in Table 3, Table 4, Table 5 and Table 6). Thus, these quantitative results are not displayed in a forest plot, but rather, are shown in Tabular format (Table 3, Table 4, Table 5 and Table 6). However, to indicate which studies contributed to the moderation analyses, Table 3, Table 4, Table 5 and Table 6 notes the reference for each study stratified by the moderator, as well as the exercise and memory temporal period.

### 3.5. Quantitative Analysis

**Figure 2:**
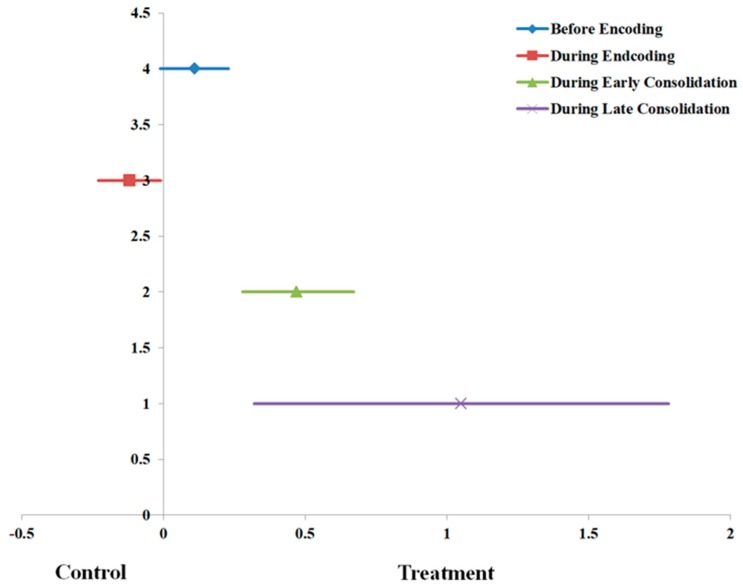
Overall pooled effect size estimates (Cohen’s d with 95% CI) across the four exercise temporal periods.

Table 3 displays the moderation results for the studies comparing exercise before memory encoding vs. control scenarios. The overall effect size for this temporal period was, d = 0.11 (95% CI: −0.01, 0.23, *p* = 0.08). This overall effect for this temporal period is also shown in Figure 2. There was evidence of a significant moderation effect for this temporal period (Q = 356.0, *df* (74), *p* < 0.001, *I*^2^ = 79.2). For young adults, acute exercise occurring before memory encoding enhanced episodic memory (d = 0.18, 95% CI: 0.06, 0.29). However, for older adults, acute exercise occurring before memory encoding impaired memory function (d = −0.53, 95% CI: −0.88, −0.18). Other significant moderators that demonstrated an enhancement effect of acute exercise prior to memory encoding included samples that utilized a mixed sex sample (d = 0.28, 95% CI: 0.14, 0.43), samples that were predominately white (d = 0.26, 95% CI: 0.02, 0.50), long-term memory outcomes (d = 0.19, 95% CI: 0.03, 0.34), vigorous-intensity exercise (d = 0.54, 95% CI: 0.19, 0.89), and cycling exercise (d = 0.46, 95% CI: 0.12, 0.81). The regression intercept for the Egger’s test (intercept = 1.34, *p* = 0.11) was not statistically significant, indicating that there was no evidence of publication bias.

Table 4 displays the moderation results for the studies comparing exercise during memory encoding vs. control. The overall effect size for this temporal period was, d = −0.12 (95% CI: −0.23, −0.01, *p* = 0.03). This overall effect for this temporal period is also shown in Figure 2. There was no evidence of a significant moderation effect for this temporal period (Q = 18.4, *df* (17), *p* = 0.36, *I*^2^ = 7.6). In this temporal paradigm, studies that included a mixed sex sample (d = −0.13, 95% CI: −0.26, 0.00) and a racially mixed sample (d = −0.27, 95% CI: −0.48, −0.06) demonstrated that acute exercise during memory encoding (vs. control) had a worse episodic memory function. Similar results occurred for long-term memory (d = −0.23, 95% CI: −0.36, −0.09) and short duration acute exercise (d = −0.20, 95% CI: −0.35, −0.04). The regression intercept for the Egger’s test (intercept = −0.90, *p* = 0.37) was not statistically significant, indicating that there was no evidence of publication bias.

Table 5 displays the moderation results for the studies comparing exercise during early consolidation vs. control scenarios. The overall effect size for this temporal period was, d = 0.47 (95% CI: 0.28, 0.67, *p* < 0.001). This overall effect for this temporal period is also shown in Figure 2. There was evidence of a significant moderation effect for this temporal period (Q = 387.2, *df* (61), *p* < 0.001, *I*^2^ = 84.2). When acute exercise occurred during the early consolidation period (vs. control), acute exercise enhanced episodic memory for young adults (d = 0.54, 95% CI: 0.35, 0.73), mixed sex samples (d = 0.60, 95% CI: 0.40, 0.80), vigorous-intensity exercise (d = 1.09, 95% CI: 0.83, 1.35), long duration acute exercise (d = 1.36, 95% CI: 1.09, 1.64), and cycling-based acute exercise (d = 1.17, 95% CI: 0.91, 1.43). Notably, the light-intensity, acute exercise during the early consolidation period (vs. control) impaired the episodic memory function (d = −0.59, 95% CI: −1.12, −0.06). The regression intercept for the Egger’s test (intercept = 3.54, *p* < 0.001) was statistically significant, indicating that there was evidence of publication bias.

Table 6 displays the moderation results for the studies comparing exercise during late consolidation vs. control scenarios. The overall effect size for this temporal period was, d = 1.05 (95% CI: 0.32, 1.78, *p* = 0.005). This overall effect for this temporal period is also shown in Figure 2. There was evidence of a significant moderation effect for this temporal period (Q = 21.2, *df* (3), *p* < 0.001, *I*^2^ = 85.9). When acute exercise occurred during the late consolidation period (vs. control), acute exercise enhanced episodic memory for long-term memory (d = 1.20, 95% CI: 0.13, 2.27), short-duration acute exercise (d = 1.31, 95% CI: 0.20, 2.43) and walking/running (d = 1.31, 95% CI: 0.20, 2.43). The regression intercept for the Egger’s test (intercept = 8.30, *p* < 0.001) was statistically significant, indicating that there was evidence of publication bias.

The summative findings across the four temporal periods are displayed in Table 7. These findings (which summarize the results from the previous tables) indicate the statistically significant positive and negative effects of acute exercise on memory across the evaluated moderators (demographic, exercise, and memory characteristics).

## 4. Discussion

Previous experimental work demonstrates that acute exercise (i.e., a structured bout of treadmill or cycling exercise) can enhance episodic memory function [15,30]. Further, as demonstrated in recent experiments [31,32,33], narrative reviews [14,35], qualitative reviews [34], and meta-analytic reviews [15,16], the timing of acute exercise appears to play a key role in subserving episodic memory function. In alignment with these previous publications, our meta-analysis demonstrates that acute exercise occurring during early memory consolidation (d = 0.47, 95% CI: 0.28, 0.67; *p* < 0.001) and during late memory consolidation (d = 1.05, 95% CI: 0.32, 1.78; *p* = 0.005) enhanced episodic memory function. These respective effect sizes represent medium and large effects. Conversely, our meta-analytic results demonstrate that acute exercise occurring during memory encoding had a negative effect on episodic memory (d = −0.12, 95% CI: −0.22, −0.02; *p* = 0.02). The temporal period involving acute exercise before memory encoding was not statistically significant at the group level (d = 0.11, 95% CI: −0.01, 0.23, *p* = 0.08), but this was driven by the statistically significant (Q = 356.0, *df* (74), *p* < 0.001, *I*^2^ = 79.2) moderation effects for various characteristics, as described below.

Of central interest of the present meta-analysis was whether study characteristics and participant attributes moderated the temporal effects of acute exercise on episodic memory function. These summative findings across the four temporal periods are displayed in Table 7. Regarding the temporal period of acute exercise occurring before memory encoding, notably, we observed an interesting age-specific effect. That is, acute exercise before memory encoding was advantageous in enhancing episodic memory function for young adults, but impaired memory function for older adults. This enhancement effect for younger adults aligns with the moderation results from the meta-analysis of Roig et al. [15]. That is, the meta-analysis of Roig et al. evaluated the effects of exercise on memory function and also evaluated various moderators (e.g., age) of this effect. We extend the meta-analysis of Roig et al. [15] by demonstrating that this age-specific effect occurs for both exercise prior to memory encoding and acute exercise during the early memory consolidation period. It is uncertain as to why older adults may have impaired episodic memory function after an acute bout of exercise. Speculatively, for older adults (vs. younger adults), acute exercise may impose greater physiological and cognitive stress, and ultimately, may impair memory function from an enhanced cognitive load effect. Similarly, it may take older adults longer to recover from an acute bout of exercise, which could have influenced these age-specific effects. Future work should conduct a side-to-side comparison of acute exercise on episodic memory among both young- and older-adults. Such work should also vary the acute exercise recovery period to determine whether this attenuates the negative memory effects observed among older adults.

Other notable findings for acute exercise before the memory-encoding period was the moderation effects for long-term memory, vigorous-intensity exercise, and cycling-based exercise. It is likely that acute exercise has a greater effect on long-term memory (vs. short-term) due to the acute exercise-induced molecular pathways that are activated to subserve long-term memory (e.g., neurotrophin production, long-term potentiation) [28,29]. We also demonstrated that vigorous-intensity, acute exercise was optimal in enhancing episodic memory, which aligns with a recent systematic review [34]. Similar to the mechanisms noted above for long-term memory, higher intensity acute exercise may more robustly modulate mechanisms (e.g., long-term potentiation) related to episodic memory function. Lastly, an interesting finding for this temporal period was that cycling-based exercise had a greater effect on enhancing episodic memory function. This was a surprising finding to us. It is expected that ambulatory exercise (vs. cycling) is a more complex movement pattern, and more complex movement patterns have greater effects on regional cerebral blood flow and cortical excitability [80,81], factors likely to subserve episodic memory [82]. However, perhaps these ambulatory activities are not more complex movement patterns than cycling, given that walking (and perhaps jogging) is part of the daily routine of the participants evaluated in these experiments. Notably, cycling may be considered a complex movement pattern, especially among novice cyclists. Future work should conduct a side-to-side comparison of ambulatory vs. cycling exercise on episodic memory function, while considering the participant’s experience with these exercise modalities. Further, particularly among novice cyclists, even a relatively light workload may be perceived as vigorous-intensity exercise. Thus, it is possible that this exercise modality-specific effect may actually be driven by an exercise intensity-specific effect. Future work is needed to help disentangle these interrelationships.

Regarding the early memory consolidation period, notable observations were that vigorous-intensity, acute exercise and long-duration acute exercise were positively associated with episodic memory function. These observations align with the mechanisms thought to enhance memory stabilization, specifically, the late-phase of long-term potentiation (discussed in detail elsewhere [28]), which is a protein synthesis-dependent process [28]. Higher-intensity and longer duration acute exercise are likely to have greater effects on the production of key proteins (e.g., brain-derived neurotrophic factor) that would subserve the late-phase of long-term potentiation [36]. In our late-phase memory consolidation temporal period analyses, our findings demonstrated that the short-duration acute exercise was advantageous in enhancing episodic memory when it occurred in this temporal period. At this point in the memory stabilization process (4-h post memory encoding), it is likely that only a short duration bout of exercise (vs. longer duration) is needed to further stabilize the memory trace.

Regarding the temporal period involving acute exercise during memory encoding, our findings demonstrated that, across various factors (e.g., mixed sex, short-duration acute exercise), acute exercise impaired episodic memory function. This finding is likely a result of reduced cognitive resources toward encoding the memory stimuli during an acute bout of exercise. Cognitive resources may be redistributed away from encoding the stimuli in order to sustain the movement itself [37,38].

When considering all four temporal periods, there was not a consistent effect of a particular moderator (e.g., young adults) across the different temporal periods. However, there were relatively few studies conducted in the early- and late-memory consolidation temporal periods; thus, we should interpret our findings accordingly. Further, we observed evidence of publication bias for both of these temporal periods, and as such, future work in these temporal periods is needed before we can definitely conclude whether or not the study and participant characteristics differentially influences episodic memory across these four temporal periods.

In addition to future work evaluating the effects of these temporal periods, as well as potential moderators, it would be a worthwhile endeavor for future work to continue to explore the mechanisms of acute exercise on episodic memory function. As discussed thoroughly elsewhere [83], exercise may help to rewire the neuronal networks involved in memory function. For example, exercise may help to coordinate neuronal firing in hippocampal circuits and enhance integration of adult-born neurons into existing hippocampal-entorhinal circuity [83]. As we recently demonstrated, exercise may enhance the functional connectivity of key memory-related brain structures (e.g., connectivity between parahippocampi and connectivity of hippocampal-orbitofrontal pathway) [84,85]. Relatedly, structural brain changes have been observed from chronic exercise [86]. From cellular and molecular perspectives, acute exercise may upregulate key neurotrophins (e.g., brain-derived neurotrophic factor) that may help facilitate neuronal communication via increasing neural activity and receptor activity [87]. Acute exercise may also be beneficial via exercise-induced increases in glucose and oxygen metabolism, as well as increases in neurotransmitter concentrations [87]. Importantly, future work should evaluate whether there are distinct mechanisms across the different exercise-memory temporal periods.

In conclusion, our meta-analytic findings demonstrate two key observations. First, the temporal period of acute exercise on episodic memory plays an important role in the exercise-memory interaction. When acute exercise occurs before memory encoding or during early and late memory consolidation, then memory enhancement effects are likely to be observed. However, when acute exercise occurs during memory encoding, memory function is likely to be impaired. Secondly, various study design and participant characteristics are likely to moderate the temporal effects of acute exercise on episodic memory function. For example, vigorous-intensity, acute exercise, and acute exercise among young adults, are likely to have greater effects when the acute bout of exercise occurs before memory encoding or during the early memory consolidation period.

## Figures and Tables

**Table 1:** Methodological quality of the included studies.

Author	Random Allocation/Counterbalance	Concealed Allocation	Baseline Comparability	Blinding of Assessors to Outcome	Between/Within Group Analysis	Reported Objective Measure of Exercise Intensity	Outcome Point Estimate	Total
Stones et al. (1993) [62]	✓		✓		✓	✓	✓	5
Schramke et al. (1997) [63]	✓		✓		✓	✓	✓	5
Labban et al. (2011) [6]	✓		✓		✓	✓	✓	5
Salas et al. (2011) [64]	✓				✓		✓	3
Nanda et al. (2013) [65]					✓		✓	2
Schmidt-Kassow et al. (2014) [10]	✓		✓		✓	✓	✓	5
Weinberg et al. (2014) [66]	✓				✓	✓	✓	4
Basso et al. (2015) [67]	✓		✓		✓	✓	✓	5
Loprinzi et al. (2015) [68]	✓		✓		✓	✓	✓	5
Bantoft et al. (2016) [69]	✓		✓		✓		✓	4
van Dongen et al. (2016) [19]	✓		✓		✓	✓	✓	5
Crush et al. (2017) [70]	✓		✓		✓	✓	✓	5
Frith et al. (2017) [31]	✓		✓		✓	✓	✓	5
Keyan et al. (2017) [71]	✓		✓		✓	✓	✓	5
Keyan et al. (2017) [72]	✓		✓		✓	✓	✓	5
McNerney et al. (2017) [73]	✓		✓		✓	✓	✓	5
Most et al. (2017) [74]	✓				✓	✓	✓	4
Sng et al. (2017) [32]	✓		✓		✓	✓	✓	5
Delancey et al. (2018) [40]	✓	✓	✓		✓	✓	✓	6
Haynes et al. (2018) [33]	✓		✓		✓	✓	✓	5
Labban et al. (2018) [75]	✓		✓		✓	✓	✓	5
Siddiqui et al. (2018) [76]	✓		✓		✓	✓	✓	5
Wade et al. (2018) [77]	✓		✓		✓	✓	✓	5
Yanes et al. (2018) [78]	✓		✓		✓	✓	✓	5
Zuniga et al. (2018) [79]	✓		✓		✓	✓	✓	5

**Table 2:** Extraction table of the evaluated studies.

Author	Sample	Study Design	Exercise Temporality	Exercise Protocol	Memory Assessment	Results
Stones et al. (1993) [62]	20 older adults, M_age_ = 84.5	Experimental; between-subject	Memory battered occurred before exercise, immediately after exercise and then 30-min post-exercise	15-min exercises, which occurred while sitting in a chair (e.g., stretching, low-intensity aerobic activity, slow rhythmical movement)	Word fluency	Exercise was associated with greater semantically cued memory (*p* < 0.01).
Schramke et al. (1997) [63]	Two age groups, each including 48 adults. Younger group, 18–38 year. Older group, 60–80 year.	Experimental; within-subject	Exercise occurred either at rest or during encoding, and similarly, either during retrieval or not.	5–7 minutes of walking in a long internal corridor.	CVLT; California verbal learning test	There was no difference in learning that was due to initial exercise condition, but both age groups showed greater recall when state was congruent before learning and delayed recall.
Labban et al. (2011) [6]	48 young adults (M_age_ = 22.0)	Experimental; between-subject	Exercise occurred before and after encoding	30-min of cycle ergometer exercise, with 20-min at moderate-intensity	Paragraph recall, with participants listening to two paragraphs and then recalling as much information as possible from the paragraphs	Exercise occurring prior to the memory task was effective in enhancing memory (*p* < 0.05).
Salas et al. (2011) [64]	80 college undergraduate students (46 women). M_age_ = 19.3, SD = 2.3	Experimental; between-subject factorial design. A 2 (encoding condition: walking vs. sitting) × 2 (retrieval condition: walking vs. sitting).	Exercise occurred either at rest or during encoding, and similarly, either during retrieval or not.	10 minutes of walking outside at a brisk pace	Word-list memory task (10 nouns presented sequentially for 6 s each)	Students who walked before encoding had significantly higher recall (M = 0.45, SD = 0.17) compared to students who sat before encoding (M = 0.36, SD = 0.15), *F*(1,76) = 6.34, ƞ^2^_p_ = 0.08.
Nanda et al. (2013) [65]	10 healthy adult male medical students. M_age_ = 19.5, SD = 0.9	Quasi-experimental; within-subject	Exercise occurred between pre- and post- memory assessments.	Cycle ergometer exercise for 30-min at moderate-intensity of 70% of heart rate reserve	Spatial span and paired associates memory task	Spatial span did not increase from pre- to post, but paired associates was significantly higher after the exercise bout.
Schmidt-Kassow et al. (2014) [10]	49 right-handed German young adults (18–30 year)	Experimental; within-subject	Exercised during encoding	Self-selected walking pace during memory encoding	40-item (Polish) word list.	Experiment 1: words recalled during walking was higher than non-walking (5.5, SD = 3.3; vs. 4.8, SD = 4.2), *F* = 6.98, *p* = 0.02, ƞ^2^_p_ = 0.31. Experiment 2: words recalled during walking was higher than non-walking (5.3, SD = 4.6; vs. 4.1, SD = 3.5), *F* = 6.44, *p* = 0.02, ƞ^2^_p_ = 0.19.
Weinberg et al. (2014) [66]	23 participants (M_age_ = 20.6 year) in the exercise group and 23 (M_age_ = 20.2 year) in the control group.	Experimental; between-subject	Exercised during early consolidation	Isokinetic dynamometer knee extension exercise. Session consisted of submaximal voluntary dynamic contractions for a warm-up, maximal voluntary isometric contractions, and 6 sets of 10 repetitions of maximal voluntary knee extension contractions. Both legs were exercised. In the control (passive) group, the experimenter passively moved the participant leg between extension and flexion.	180 images from the IAPS. Follow-up memory recall assessment took place 48-h later. The retrieval task included 90 studied images and 90 new images. Participants were instructed to indicate “remember”, “familiar”, or “new” after seeing each image.	There was no *valence × group* interaction effect. There was a main effect for valence in that participants remembered more positive and negative images than neutral images.
Basso et al. (2015) [67]	85 young adults, M_age_ = 22.1	Experimental; between-subject	Memory tasks occurred before exercise and at various time-points after exercise (30–120 min)	50-min of vigorous-intensity exercise on cycle ergometer	Hopkins verbal learning test revised, modified Benton visual retention test, Digit span	Acute exercise improved prefrontal-cortex, but not hippocampal-dependent memory function.
Loprinzi et al. (2015) [68]	87 young adults, M_age_ = 21.4 year	Experimental; between-subject	Exercise before memory task	Light, moderate, and vigorous exercise	Spatial span and paired associates	Acute exercise was not associated with either memory outcome.
Bantoft et al. (2016) [69]	45 undergraduate students, M_age_ = 22.6 year (6.2)	Experimental; within-subject	Sitting, standing or walking during memory task	Low-intensity walking	Digit span	There were no differences in memory performance across the three conditions.
van Dongen et al. (2016) [19]	72 young adults, approximately 22 years	Experimental; between-subject	Exercise immediately after encoding and 4 hours after encoding	35 min of intermittent high-intensity exercise on cycle ergometer	Paired associates learning task	Exercising 4 hours after memory encoding was advantageous in improving memory function.
Crush et al. (2017) [70]	352 participants, mean age approximately 21 years	Experimental; between-subject	Exercise occurring before memory assessment	16 total groups, with groups ranging from 10 min of exercise to 60 min of exercise, including resting periods of either 5, 15, or 30 min	Spatial span	Shorter exercise recovery periods had a greater effect on memory performance.
Frith et al. (2017) [31]	88 participants (22 per group), approximate age = 21 years.	Experimental; between-subject	Exercise occurring before, during, and after memory encoding	15-min treadmill bout of progressive high-intensity aerobic exercise	RAVLT	High-intensity exercise prior to memory encoding was effective in enhancing long-term memory, for both 20-min delay (*F* = 3.36, *p* = 0.02, ƞ^2^_p_ = 0.11) and 24-h delay (*F* =2.80, *p* = 0.04, ƞ^2^_p_ = 0.09).
Keyan et al. (2017) [71]	49 undergraduates between 18–29 years	Experimental; between-subject	Exercise occurred during the early memory consolidation period	Stepping exercise for 10-min on a 15 cm stepper, with a goal of exercising at 50%–85% of max.	Viewed a film depicting a car accident. Involves 10 min of live footage depicting emergency workers attending the scene of a motor vehicle accident.	Exercise (vs. control) did not induce more recall of central (*t* = 0.11, *p* > 0.05) or peripheral (*t* = 0.42, *p* > 0.05) details of the accident film. However, those that exercise recalled more intrusive memories of the car accident (*t* = 2.36, *p* = 0.02, d = 0.68).
Keyan et al. (2017) [72]	54 healthy undergraduate students, M_age_ = 19.5 (3.0)	Experimental; between-subject	During a memory reconsolidation paradigm, participants either exercised or did not exercise after memory reactivation	20–25 min of incremental cycling	Trauma film depicting the aftermath of a highway car crash	The exercise with reactivation condition recalled more central details of the trauma film.
McNerney et al. (2017) [73]	Experiment 1: 136 young adults, M_age_ = 19.2 (1.2) Experiment 2: 132 young adults, M_age_ = 19.1 (1.2)	Experimental; between-subject	Exercise occurring before and after memory encoding	2-min of sprints	Paired associate learning, procedural learning, and text memory	Improvements in procedural and situation model memory occurred, regardless of whether exercise occurred before or after memory encoding.
Most et al. (2017) [74]	Experiment 1: 82 undergraduate psychology students (M_age_ = 19.9). Experiment 2: 83 undergraduate psychology students (M_age_ = 19.9). Experiment 3: 48 undergraduate psychology students (M_age_ = 19.2). Experiment 4: 75 undergraduate psychology students (M_age_ = 21.1).	Experimental; between-subject	Exercise occurring after memory encoding	5-min of step exercise	Paired faces and names.	Acute exercise in the early consolidation period enhanced memory.
Sng et al. (2017) [32]	88 participants, approximately 21–25 years (mean for each group)	Experimental; between-subject	Exercise occurred before, during and immediately after memory encoding	15-min moderate intensity brisk walking (self-selected)	RAVLT	Exercising before memory encoding was superior for enhancing learning (*p* = 0.05), 24-h memory recognition (*p* = 0.05) and 24-h memory attribution (*p* = 0.006).
Delancey et al. (2018) [40]	40 participants, approximately 20 years of age	Experimental; between-subject	Exercise occurring 4 hours after memory encoding	High-intensity bout of exercise for 15 minutes	RAVLT	Those who exercise during the consolidation period have a greater 24-h follow-up memory attribution (*p* = 0.04).
Haynes et al. (2018) [33]	24 participants (M_age_ = 20.9; SD = 1.9), with 66.7% being female.	Experimental; within-subject	Exercise occurring before, during, and after memory encoding	Self-selected brisk walking pace for 15-min	RAVLT	Short-term memory was greater in the visit that involved exercise prior to the memory task (*F*= 3.76, *p* = 0.01, ƞ^2^_p_ = 0.79). Similar results occurred for long-term memory, but there were no exercise effects on prospective memory.
Labban et al. (2018) [75]	15 Participants; M_age_ = 22.7, SD = 3.1	Experimental; within-subject	Exercise occurring both before and after memory encoding.	30-min of moderate intensity cycling	RAVLT	Exercise that occurred before encoding (vs. control) was advantageous in enhancing long-term memory, including both 60-min delayed memory (*p* = 0.03) and 24-h delayed recall (*p* = 0.03).
Siddiqui et al. (2018) [76]	20 participants (60% male). M_age_ = 21.1; SD = 1.0	Experimental; within-subject	Exercise occurring both before and during memory encoding.	20-min treadmill walk at a self-selected brisk walking pace	The Deese-Roediger-McDermott (DRM) paradigm. Included a 15-item word list.	For both short-term and long-term memory, the visit the involved exercise before the memory task resulted in the greatest memory performance (*F* = 11.56, *p* < 0.001, ƞ^2^_p_ = 0.38)
Wade et al. (2018) [77]	34 female participants; M_age_ = 20.5 (1.2) in the exercise group and 20.8 (1.8) in the control group.	Experimental; between-subject	Exercise occurred before memory encoding	15-min treadmill walk at a self-selected brisk walking pace	Emotional memory assessment using images from the IAPS (International Affective Picture System).	There were no statistically significant group differences across any of the assessment periods (i.e., 1-day, 7-day, and 14-day follow-up assessments).
Yanes et al. (2018) [78]	40 participants, M_age_ = 21.0	Experimental; between-subject	Exercise occurred before memory encoding	15-min treadmill walk at a self-selected brisk walking pace	6-paragraph passage for memory recall	Exercise before encoding had greater scores on the short-term and long-term memory assessments, but this did not reach statistical significance (*F* = 1.0, *p* = 0.32, ƞ^2^_p_ = 0.03).
Zuniga et al. (2018) [79]	Experiment 1 (*N* = 30), M_age_ = 20.4 (1.8); Experiment 2 (*N* = 57), M_age_ = 20.6 (4.1) in low-fit group and M_age_ = 19.4 (1.6) in high-fit group.	Experimental; within-subject	Exercise occurred before memory encoding	3-min warm-up period on the treadmill, followed by 10-min of walking at either light or moderate-intensity.	Three lists of 30 concrete English nouns from the MRC Psycholinguistic database.	Both light-intensity (*t* = 2.79, *p* = 0.01) and moderate-intensity (*t* = 3.02, *p* = 0.006) recalled more words than the sedentary condition. Results were similar when comparing high-fit to low-fit individuals.

CVLT; California verbal learning test; IAPS, International Affective Picture System; RAVLT, Rey Auditory Verbal Learning Task.

**Table 3:** Moderation results for exercise before memory encoding vs. control.

Moderator	Exercise Before Memory Encoding vs. Control				
	Reference	Number of Effect Size Contributions	Effect Size (Cohen’s d)	Lower CI	Upper CI
**Age**					
Young Adult	[6,31,32,33,63,64,65,68,70,71,72,73,74,75,76,77,78,79]	66	0.18 *	0.06 *	0.29 *
Older Adults	[62,63]	9	−0.53 *	−0.88 *	−0.18 *
**Sex**					
Male	[65]	2	0.32	−0.41	1.05
Female	[77]	3	−0.14	−0.75	0.46
Mixed	[6,31,32,33,64,68,71,72,73,74,75,76,78,79]	42	0.28 *	0.14 *	0.43 *
Predominately Female	[62,70,79]	22	−0.06	−0.27	0.15
**Race-Ethnicity**					
Predominately white	[33,68,76,78,79]	17	0.26 *	0.02 *	0.50 *
Mixed	[6,31,32,70,75,77,79]	31	0.10	-0.08	0.29
**Memory Type**					
Short-term	[31,32,33,65,70,73,74,76]	23	−0.01	−0.22	0.21
Long-term	[6,31,32,33,62,63,64,71,72,73,75,76,77,78,79]	46	0.19 *	0.03 *	0.34 *
**Exercise Intensity**					
Light	[62,63,68,79]	17	−0.20	−0.45	0.04
Moderate	[6,32,33,64,68,70,73,74,75,76,77,78,79]	45	0.14	−0.01	0.28
Vigorous	[31,65,68,71]	9	0.54 *	0.19 *	0.89 *
**Exercise Duration**					
Short	[31,32,33,62,63,64,70,71,73,74,77,78,79]	45	0.07	−0.09	0.22
Medium	[6,65,68,70,72,75,76]	24	0.20	−0.02	0.41
Long	[70]	6	0.04	−0.37	0.45
**Exercise Modality**					
Walking/Running	[31,32,33,63,64,68,70,73,76,77,78,79]	57	0.06	−0.07	0.19
Cycling	[6,65,72,75]	10	0.46 *	0.12 *	0.81 *

* indicates statistically significant effect size (*p* < 0.05).

**Table 4:** Moderation results for exercise during memory encoding vs. control.

Moderator	Exercise During Memory Encoding vs. Control				
	Reference	Number of Effect Size Contributions	Effect Size (Cohen’s d)	Lower CI	Upper CI
**Sex**					
Mixed	[10,31,32,33,76]	16	−0.13 *	−0.26 *	0.00 *
Predominately Female	[69]	2	−0.09	−0.33	0.15
**Race-Ethnicity**					
Predominately white	[33,76]	6	0.00	−0.17	0.17
Mixed	[31,32]	8	−0.27 *	−0.48 *	−0.06 *
**Memory Type**					
Short-term	[31,32,33,69,76]	6	0.02	-0.14	0.18
Long-term	[10,31,32,33,76]	12	−0.23 *	−0.36 *	−0.09 *
**Exercise Intensity**					
Light	[10,69]	4	−0.15	−0.35	0.05
Moderate	[32,33,76]	10	−0.09	−0.26	0.07
Vigorous	[31]	4	−0.18	−0.49	0.14
**Exercise Duration**					
Short	[31,32,33]	12	−0.20 *	−0.35 *	−0.04 *
Medium	[10,76]	4	0.00	−0.22	0.22

* indicates statistically significant effect size (*p* < 0.05).

**Table 5:** Moderation results for exercise during early consolidation vs. control.

Moderator	Exercise During Early Consolidation vs. Control				
	Reference	Number of Effect Size Contributions	Effect Size (Cohen’s d)	Lower CI	Upper CI
**Age**					
Young Adult	[6,19,31,32,33,63,64,67,73,74,75]	59	0.54 *	0.35 *	0.73 *
Older Adults	[63]	3	−0.95	−1.76	−0.15
**Sex**					
Mixed	[6,19,31,32,33,64,67,73,74,75]	55	0.60 *	0.40 *	0.80 *
**Race-Ethnicity**					
Predominately white	[33]	4	−0.07	−0.76	0.63
Mixed	[6,31,32,75]	12	−0.14	−0.56	0.28
**Memory Type**					
Short-term	[31,32,33,67,73,74]	35	1.05 *	0.79 *	1.30 *
Long-term	[6,19,31,32,33,63,64,73,74,75]	26	−0.14	−0.40	0.11
**Exercise Intensity**					
Light	[63]	6	−0.59 *	−1.12 *	−0.06*
Moderate	[6,32,33,64,73,75]	19	−0.02	−0.31	0.27
Vigorous	[19,31,67]	33	1.09 *	0.83 *	1.35 *
**Exercise Duration**					
Short	[31,32,33,63,64,73,74]	29	−0.13	−0.34	0.09
Medium	[6,19,75]	5	0.21	−0.32	0.74
Long	[67]	28	1.36 *	1.09 *	1.64 *
**Exercise Modality**					
Walking/Running	[31,32,33,63,64,73,75]	26	−0.19	−0.43	0.05
Cycling	[6,19,67]	32	1.17 *	0.91 *	1.43 *

* indicates statistically significant effect size (*p* < 0.05).

**Table 6:** Moderation results for exercise during late consolidation vs. control.

Moderator	Exercise During Late Consolidation vs. Control				
	Reference	Number of Effect Size Contributions	Effect Size (Cohen’s d)	Lower CI	Upper CI
**Memory Type**					
Long-term	[19,40]	3	1.20 *	0.13 *	2.27 *
**Exercise Duration**					
Short	[40]	3	1.31 *	0.20 *	2.43 *
**Exercise Modality**					
Walking/Running	[40]	3	1.31 *	0.20 *	2.43 *

* indicates statistically significant effect size (*p* < 0.05).

**Table 7:** Summative findings of the moderation results across the four acute exercise and memory temporal periods.

	Exercise and Memory Temporal Periods			
Moderator	Before vs. Control	During vs. Control	Early vs. Control	Late vs. Control
**Demographic Characteristic**				
Young adults	**+**		**+**	
Older adults	**−**			
Mixed-sex sample	**+**	**−**	**+**	
Predominately white	**+**			
Racially-Ethnically mixed sample		**−**		
**Exercise Characteristic**				
Light-intensity			**−**	
Vigorous-intensity	**+**		**+**	
Short-duration		**−**		**+**
Long-duration			**+**	
Cycling	**+**		**+**	
**Memory**				
Short-term memory			**+**	
Long-term memory	**+**	**−**		**+**

The four temporal periods included: (1) exercise before memory encoding vs. control, (2) exercise during memory encoding vs. control, (3) exercise during early consolidation vs. control, and (4) exercise during late consolidation vs. control. +, statistically significant positive effect, −, statistically significant negative effect.
